# A Novel Technique Enables Quantifying the Molecular Interaction of Solvents with Biological Tissues

**DOI:** 10.1038/s41598-019-45637-7

**Published:** 2019-06-27

**Authors:** Sakshi Yadav, Semih Gulec, Rafael Tadmor, Ian Lian

**Affiliations:** 1 Dan F. Smith Department of Chemical Engineering, Lamar University, Beaumont, TX 77710 USA; 2 Department of Mechanical Engineering, Ben-Gurion University of the Negev, Beer-Sheva, 8410501 Israel; 3 Department of Biology, Lamar University, Beaumont, TX 77710 USA

**Keywords:** Permeation and transport, Chemical engineering, Materials science

## Abstract

The pharmaceutical industry uses various solvents to increase drug penetrability to tissues. The solvent’s choice affects the efficacy of a drug. In this paper, we provide an unprecedented means of relating a solvent to a tissue quantitatively. We show that the solvents induce reorientation of the tissue surface molecules in a way that favors interaction and, therefore, penetrability of a solvent to a tissue. We provide, for the first time, a number for this tendency through a new physical property termed Interfacial Modulus (*G*_*s*_). *G*_*s*_, which so far was only predicted theoretically, is inversely proportional to such interactions. As model systems, we use HeLa and HaCaT tissue cultures with water and with an aqueous DMSO solution. The measurements are done using Centrifugal Adhesion Balance (CAB) when set to effective zero gravity. As expected, the addition of DMSO to water reduces *G*_*s*_. This reduction in *G*_*s*_ is usually higher for HaCaT than for HeLa cells, which agrees with the common usage of DMSO in dermal medicine. We also varied the rigidities of the tissues. The tissue rigidity is not expected to relate to *G*_*s*_, and indeed our results didn’t show a correlation between these two physical properties.

## Introduction

Drug delivery via transdermal, intramuscular and subcutaneous injections are often performed instead of oral administration, especially for administering therapeutic peptides or proteins^1,2^. Water is a commonly used medium for transdermal drug delivery because tissue hydration appears to increase transdermal delivery of both hydrophilic and lipophilic permeants^3–5^. However, it is not a universal medium because it does not increase the drug percutaneous absorption^6^. Thus, in many cases, there is a desire to increase the efficacy of drug delivery^7^. One long-standing approach to increase drugs penetrability to the skin has been to use *penetration enhancers* that interact with skin constituents to promote drug flux. Most commonly used enhancers are water, azone, pyrrolidones, fatty acids, alcohols, and sulfoxides of which Dimethyl sulfoxide (DMSO) is the most prevalent example^3,8–10^. Sulfoxides in general, and DMSO in particular, have been proven to be better at transdermal drug delivery compared to water^3,8–13^.

DMSO is a common drug solvent for *in vitro* and *in vivo* applications primarily due to its enhanced solubility for pharmaceutical reagents, since many drugs drugs are not soluble in hydrophilic solvents^1,2,14^. It is also used as an anti-freeze agent (cryo-preservation)^15^. In addition of being an effective solvent for small molecules, DMSO is also capable of dissolving macromolecules such as peptides and proteins^1,2^ and facilitating drug diffusion across cell membranes comprised of lipid bilayers. Chemically, it is a powerful aprotic solvent which hydrogen bonds with itself rather than with water; it is colorless, odorless and hygroscopic and is often used in many areas of pharmaceutical sciences as a “universal solvent”^3^. Moreover, it is cost effective to synthesize, is stable at room temperature conditions and has a workable cytotoxicity of up to 10% for most biomedical purposes.

In this paper we quantify DMSO’s tissue penetrative capacity when mixed with water. This penetrative capacity of the solvent influences its wetting behavior on the substrates and effect the drop pinning. For instance, HaCaT substrate represents cell line from an adult human skin and since skin acts as a barrier to penetrating molecules, chemical permeability enhancers such as DMSO are added to deliver active molecules into or via the skin^10–13^.

Though a methodic choice of solvents is important for the drug delivery purposes, and there are studies that target the various parameters in the problem, still the solvent penetration efficacy is not quantifiable. For example, it is known that the lipids of the topmost layer of the skin, the *stratum corneum*, are the main barrier to penetration of exogenous substances through the skin^1,10^. Yet, evaluating the degree of enhancement of such agents currently lacks a quantitative method. In this paper we show a method for quantifying penetration enhancers, and demonstrate it on DMSO and water. We study the interaction of the surface molecules of HeLa and HaCaT tissue cultures with water and how addition of DMSO to water can influence this interaction. HaCaT substrates are cell line from an adult human skin and HeLa substrates are cervical tumor cell line. Rigidities of HeLa and HaCaT cell substrates are chosen such that they mimic their respective *in vivo* conditions while water and DMSO are chosen for their pharmaceutical applications. Both solvents are known to not interfere with various drugs and can efficiently interact and penetrate tissues along with other active agents^10–13^. To quantify the way addition of DMSO to water enhances the tissue penetrative capacity of drugs^8–13^, we use the concept of the interfacial modulus (*G*_*s*_) as explained below.

## Theoretical Background

**Figure 1:**
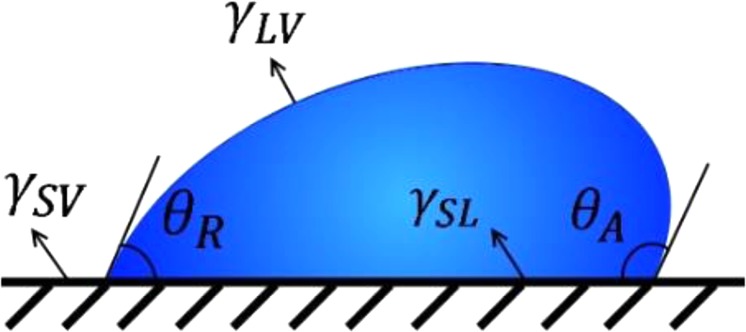
Some drop parameters used in this study are shown while a droplet is sliding on a solid surface.

The approach we use to determine the onset of drop motion is based on Shanahan, de Gennes and Tadmor model^16–19^ and assumes surface deformation. This approach is particularly sensitive to the normal force that acts on the drop, and therefore requires measurements at effective zero gravity. However, it allows the determination of the interfacial modulus according to:

1 $${f}_{\parallel }=\frac{4{\gamma }_{LV}^{2}\,\sin \,\theta }{{G}_{s}}(\cos \,{\theta }_{R}-\,\cos \,{\theta }_{A})$$

where, *f*_∥_ is the lateral force required to slide the drop along the surface, *γ*_*LV*_ is the liquid-vapor surface tension, *θ* is the contact angle that the drop adopted when it was resting on the surface before the onset of motion, *θ*_*R*_ and *θ*_*A*_ are the drop receding and advancing contact angles, respectively as shown in Fig. 1 and *G*_*s*_ (the interfacial modulus) represents the tendency of the surface to resist interacting with the liquid. It can, therefore, serve as a measure of solvent’s affinity to a certain substrate^17^. The determination of *G*_*s*_ is done through measurements of drop retention forces at effective zero gravity, i.e. at zero normal force. The force considered in Eq. (1) is taken at the onset of sliding.

This approach considers outmost surface layer deformation which is proportional to the Laplace pressure inside the drop and occurs in the normal direction (the direction *γ*_*LV*_ sin*θ* pulls on the surface^18,20–23^). The normal component, *γ*_*LV*_ sin*θ* creates a ridge at the triple line, that although doesn’t affect the macroscopic contact angle, it does lower the rate of liquid wetting the surface, makes solids exhibit contact angle hysteresis, can increase the drop retentive force and exhibit *time effect*^21,23^ at solid-liquid interface.

To experimentally determine force for the onset of motion of a sliding drop at zero normal force, we use Centrifugal Adhesion Balance (CAB)^20,21,23^ which is described in the next section.

## Materials and Method

**Figure 2:**
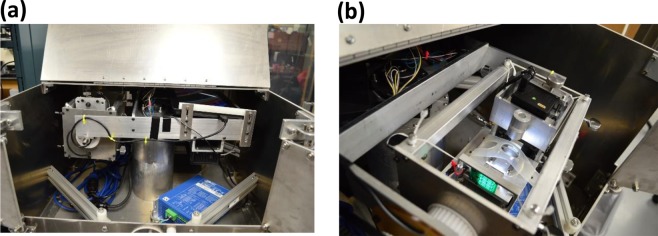
Pictures of CAB model CAB15G14. (**a**) Centrifugal arm of CAB. (**b**) Goniometer inside the CAB.

**Figure 3:**
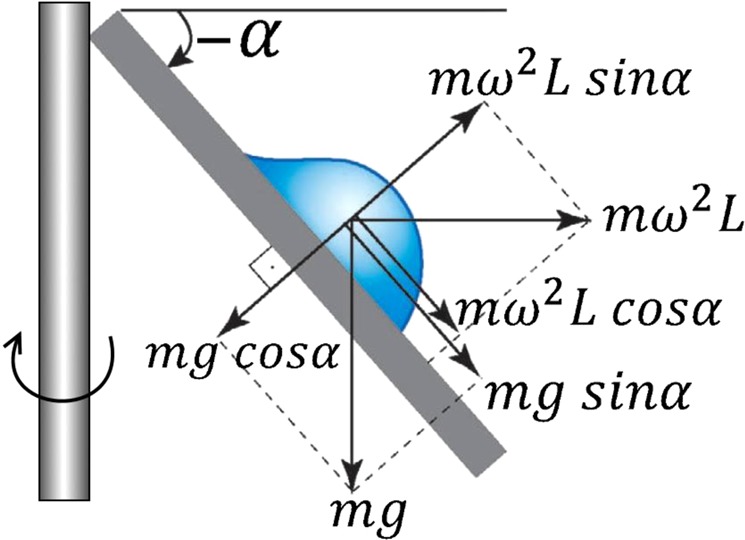
Schematics for CAB alignment for drop sliding at zero normal force.

To study *time effect*, we study lateral adhesion of a drop after allowing it to rest undisturbed for a fixed *waiting time* (*t*_*still*_) duration. We use the Centrifugal Adhesion Balance (CAB)^20,21,23–26^ so that the normal components of the gravitational and centrifugal forces cancel each other, namely a state of zero normal force (i.e. effective zero gravity), and their lateral components are gradually increased. Figure 2 shows the working parts of CAB, and the schematics in Fig. 3 shows the vectorial summation from which one can derive the equations for the normal and lateral forces: Eq. (2) for the lateral and Eq. (3) for the normal forces.

Figure 3 shows the schematics of forces acting on a drop placed in the CAB goniometer, which is located at the end of the centrifugal arm. CAB allows to measure forces and obtain visual drop data (drop diameter, height, contact angle etc.) and store it in a nearby computer.

CAB manipulates normal and lateral forces according to following equations:

2 $${f}_{\parallel }=m({\omega }^{2}R\,cos\alpha -g\,sin\alpha )$$

3 $${f}_{\perp }=m({\omega }^{2}\,R\,sin\alpha +g\,\cos \,\alpha )$$

where *f*_⊥_ and *f*_∥_ are the normal and lateral force acting on the drop, respectively, *m* is the drop’s mass, *ω* is the CAB angular velocity, *R* is the drop’s distance from the CAB’s center of rotation, *g* is the gravitational acceleration, and *α* is the tilt angle with respect to the horizon. For the experiments done in this study, *f*_⊥_ = 0 was maintained and *f*_∥_ was gradually increased. The substrates used were HeLa and HaCaT tissue culture cells.

Culture human cell lines HaCaT and HeLa (both are epithelial tissue cells) were obtained from the American Type Culture Collection (ATCC). The cells were seeded on silicone plates mimicking physiological tissue stiffness with rigidity of 2 kPa, 8 kPa and 64 kPa (manufactured by MuWells inc) and maintained in DMEM supplemented with 10% fetal bovine serum at the condition of 37 °C and 5% CO_2_. After reaching 100% confluency, cell samples were crosslinked and preserved in 4% formaldehyde for subsequent experiments. Liquid solution used are deionized (DI) water (treated with Barnstead Nanopure Purification system, specific conductance (25 °C) ≤ 0.7 × 10^−6^ Ω^−1^ cm^−1^) and 10% DMSO-90% water solution (DMSO supplied by Sigma Aldrich, ≥99.5% GC) for their biological significance.

The cell cultures used are cleaned and dried by a DI water rinse followed by 70% (30% DI water) ethanol rinse and finally a 90% (10% DI water) ethanol rinse. This step is crucial since biological tissues are naturally wet, both water and DMSO aqueous solutions would wet them completely and contact angles couldn’t be measured. Therefore, to measure *G*_*s*_, there is a need to have the tissue in a dry form so that its outer side is more hydrophobic, and its more hydrophilic functional groups are buried inside the layer. One way to achieve this, is to expose the tissue to air over a long time. A quicker way is DI water and ethanol rinse as explained above.

**Figure 4:**
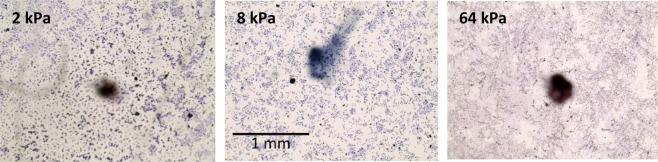
Microscopic images of HeLa samples of different rigidities. The blue specs on the substrate are the HeLa cells and the black spot is not part of the system (it is a mark underneath the petri dish that helps centering of the sample).

Extra care was taken to prevent the contamination of the tissue samples or alter their surface properties due to human error. However, it is important to note that even in a controlled environment the cell distribution in the substrate cannot be controlled and has a random nature (shown in Fig. 4). Consequently, placing the drops at different locations on the same sample, or different samples, results in different drop retention forces and exhibit large errors in the measured data.

## Results and Discussions

**Figure 5:**
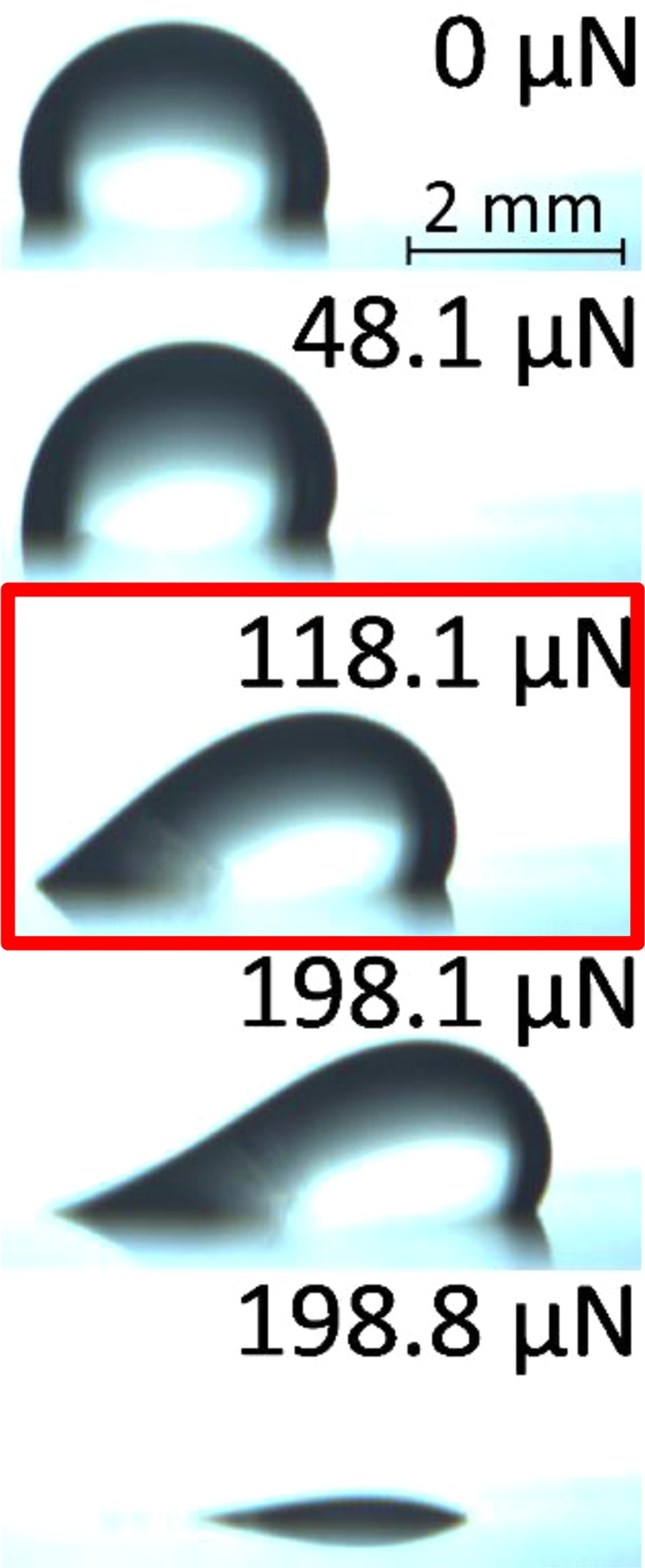
Pictures taken during a CAB run with 5 μl water drop on HeLa 64 kPa at zero normal force and growing lateral forces as noted in the figure. The image highlighted with the red box represents the moment of onset of drop detachment considered to determine the lateral retention force. The numbers on the top of the frames represent the lateral force on the drop at the time the frames were taken. Note, the onset of motion was determined from the onset of detachment of the rear end of the drop.

To determine *G*_*s*_ of substrate-solvent systems, we need to determine their lateral retention forces (*f*_∥_ in Eq. (2)). The drops were placed on the surface at zero velocity and were left undisturbed for *t*_*still*_ and then subjected to an increasing lateral force at zero normal force until their rear edge begins to move (as shown in Fig. 5). This was done for 7 different resting periods on 7 different spots on a sample (experiments were repeated on several different samples of each stiffness).

**Figure 6:**
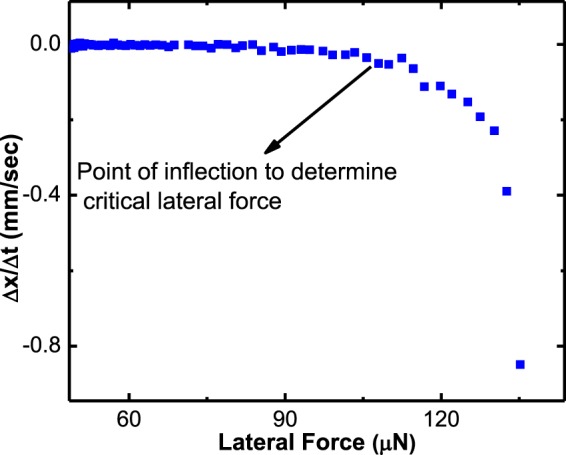
The change in drop position (receding edge) with time (Δ*x*/Δ*t*) versus lateral force, during a CAB run of effective zero gravity. The data points shown are for the whole duration the CAB was active.

Figure 5 shows the selected images from a typical experimental run of a water drop detachment from 64 kPa HeLa substrate. The drop is subjected to a gradually increasing lateral force until its rear edge de-pins and begins to move. That moment is considered as drop onset of motion and the lateral force that causes the drop to de-pin is called lateral retention force. This precise moment can be determined by observing the change in drop position with time. Figure 6 shows a plot of the change in the position of the drop’s receding edge with time including the point of inflection from which the lateral retention force was determined. The *G*_*s*_ values of each substrate - liquid pair are measured from values taken at that moment.

**Figure 7:**
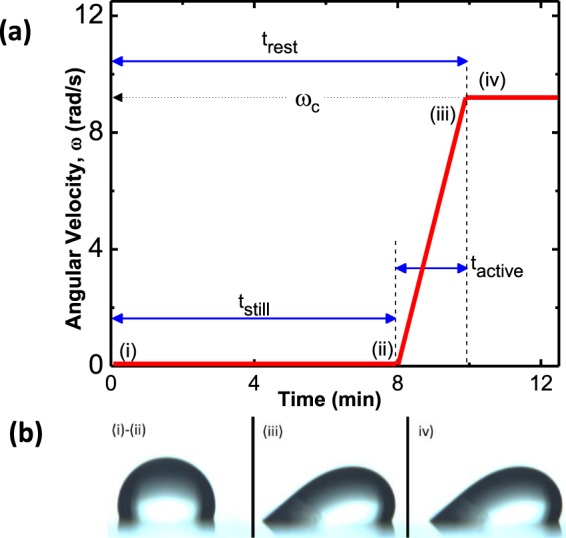
(**a**) The variation of the angular velocity (*ω*), during the measured time. The drop is allowed to rest in the stationary CAB for a prescribed period, *t*_*still*_, after which *ω* is gradually increased until, at a certain critical value, the drop starts sliding along the surface. The drop is pinned to the surface from right after placement until just before the critical *ω* is reached; this whole time is termed *t*_*rest*_. (**b**) Drop pictures as taken at different stages of the measurement. From (i) to (ii) no lateral force is applied and the drop is symmetric and pinned to the surface; during the active stage, it is deformed as shown in (iii) but it is still pinned to the surface. Once the critical *ω* is reached the drop slides as shown in (iv).

**Figure 8:**
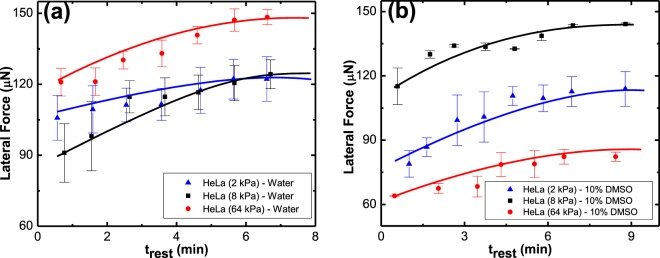
The lateral retention force, *f*_∥_, required to slide a 5 µl (**a**) DI water drop and (**b**) 10% DMSO drop on various rigidities of HeLa substrates as a function of resting time, *t*_*rest*_, at zero normal force.

As can be seen in Fig. 6, there are many data points at the beginning of the run (small lateral forces). The reason for this is the nature of the experiment that consist of a time the drop is waiting on the surface as described in Fig. 7. The first part of the waiting time is a still time, *t*_*still*_, during which the drop is resting motionless on the substrate and the CAB is still. After that, the CAB’s arm starts rotating, the sliding force on the drop increases and the drop is still motionless while maintaining zero effective gravity. The time that elapses from the moment the CAB’s arm started rotating till the moment the drop started to slide is noted as *t*_*active*_. Thus, the total time that the drop is resting on the surface prior to sliding, *t*_*rest*_, is *t*_*rest*_ = *t*_*still*_ + *t*_*active*_. Figure 8 shows CAB measurements of *f*_∥_ required to slide a drop as a function of drop resting time (*t*_*rest*_).

**Figure 9:**
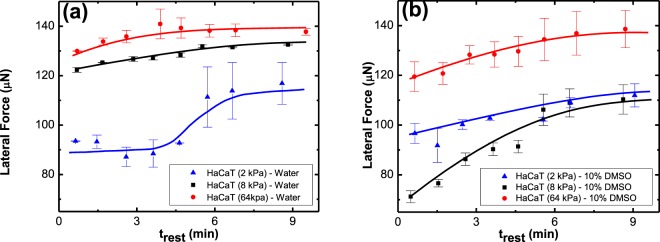
Lateral retention force, *f*_∥_, required to slide a 5 µl (**a**) DI water and (**b**) 10% DMSO drop on HaCaT substrate as a function of resting time, *t*_*rest*_, at zero normal force.

Figure 8 shows lateral retention force, *f*_∥_, required to slide water and aqueous DMSO on HeLa tissue cultures. The common feature in Fig. 8 is that the retention force, required to set a drop in motion, increases as a function of *t*_*rest*_. With the increase in resting time, the retention force increases (i.e. *df*_∥_/*dt*_*rest*_ ≥ 0 and generally ${d}^{2}{f}_{\parallel }/d{t}_{rest}^{2}\le 0$ for *t*_*rest*_ ≥ 0) and reaches, or clearly approaches, a plateau. This dependence of retention force on resting time or *time effect* is observed for HaCaT-Water and HaCaT-10% DMSO system as well (Fig. 9).

We reiterate that, extra care was taken to prevent the contamination of the tissue samples or alter their surface properties due to human error. However, it is important to note that even in a controlled environment the cell distribution in the substrate cannot be controlled and has a random nature (shown in Fig. 4). Consequently, placing the drops at different locations on the same sample, or different samples, results in different drop retention forces and exhibit large errors in the measured data.

**Figure 10:**
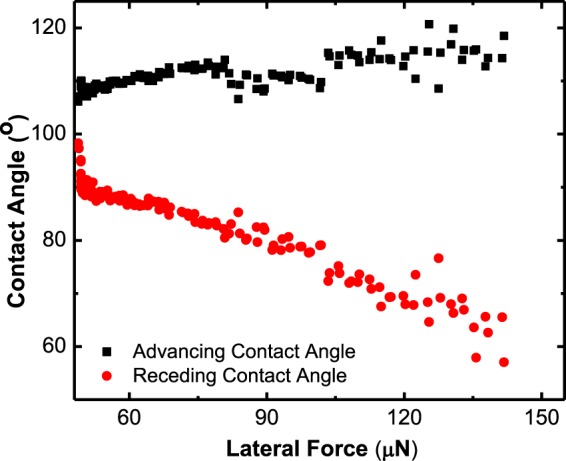
Change in the receding and advancing contact angles of a 5 µl DMSO drop on 8 kPa HaCaT substrate with gradually increasing lateral force and effective zero gravity.

The gradual increase in lateral force causes the drop position to change with time and also changes the drop receding and advancing contact angles as shown in Fig. 10. We observed a higher variation in receding edge than advancing edge as the lateral force increases.

**Figure 11:**
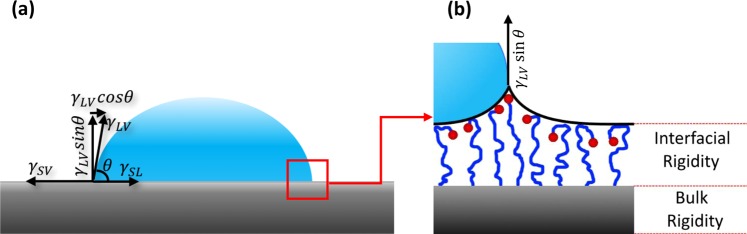
(**a**) A 2D representation of a drop on a surface describing the interfacial tensions as force vectors per length. These forces are balanced along the x axis giving rise to the Young equation. Along the vertical axis there is a normal component that takes a long time to be balanced as the solid molecules slowly reorient and deform. (**b**) Schematic diagram of molecular deformation at the triple line for a case in which the end group favors interaction with the solvent, and the main chain favors interaction with the air. The resistance of the solid molecules to deform in a way that will increase the solid-liquid interaction, is described by the interfacial modulus, *G*_*s*_.

Figures 8 and 9 show the variation of the forces as a function of the time that the drop was resting, motionless, on the surface, before sliding. This phenomenon can be theoretically linked to the gradual nature of surface deformation and molecular reorientation as illustrated in Fig. 11.

At the triple line, there are stresses due to the normal component of the surface tension: *γ*_*LV*_ sin*θ* (see Fig. 11). Based on Shanahan, de Gennes and Tadmor model^16–19,21,23,27^, these stresses result in a deformation of the solid’s outmost layer, as shown in Fig. 11b. This deformation is usually topographically negligible and as the time progresses it is balanced out with solid surface molecule reorientation. The solvents induce reorientation of the tissue surface molecules in a way that it increases the solid-liquid interaction. This reorientation takes time, as functional groups that are buried in the substrate, slowly make their random walk to the interface. The reason for the system to prefer this migration to the interface is the potential for a lower interfacial energy, i.e. a stronger intermolecular interaction, that results from the introduction of the solvent to the substrate. The increase of the solid- liquid intermolecular interaction results in an increase in the retention force seen in Figs 8 and 9. The force slowly increases with time until it reaches a plateau when the rate at which the random walk to the interface and away from it equalizes.

**Figure 12:**
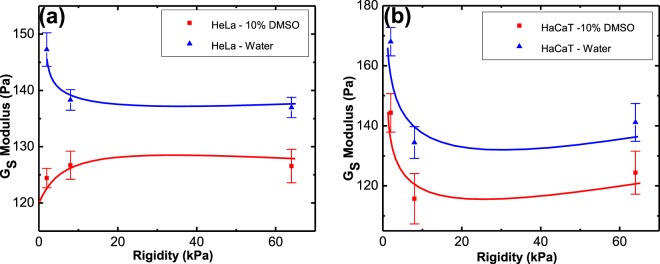
*G*_*s*_ as a function of varying rigidity of (**a**) HeLa and (**b**) HaCaT, for water and 10% DMSO drop. Note, that the different rigidities represent different surfaces, and no trend is expected as a function of the rigidity. Rather, this plot aims to show that adding DMSO to water causes a reduction in *G*_*s*_ and exemplify it for different surfaces.

**Figure 13:**
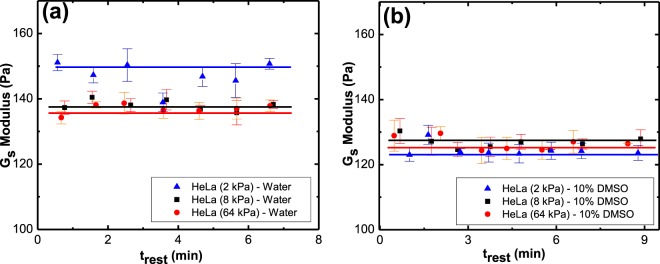
*G*_*s*_ of HeLa with (**a**) water and (**b**) aqueous DMSO as solvents as a function of drop resting time.

The interfacial modulus, *G*_*s*_, quantifies the resistance of the solid surface’s molecules to interact with liquid’s molecules. It relates to the intermolecular interaction between the substrate and the solvent only at the outmost layer of the solid. In Fig. 12 we plot the values of *G*_*s*_ for the various tissues used in this study. Figure 12 shows that the addition of DMSO to water reduces *G*_*s*_ for both HeLa and HaCaT cells. This reduction does not have any relation to the substrate bulk rigidity and is only a function of interaction between solvent and interfacial rigidity. The surface rigidities correlate to the bulk phase of solids in this work, whereas *G*_*s*_ relates to the interfacial phase. Therefore, the rigidity of the bulk phase is not expected to correlate to the *G*_*s*_ values. However, each rigidity has its own unique surface properties that results in its own unique *G*_*s*_ value. A schematic representation for “interfacial rigidity” and “bulk rigidity” in Fig. 11 is given to visualize the difference for better understanding. Addition of DMSO to water reduces the *G*_*s*_ value by 15% for 2 kPa HeLa and 8% for 8 kPa and 64kPa HeLa whereas for HaCaT, for the same rigidities, the reduction is by 14%, 14% and 12%, respectively. Note, in our system, the interfacial modulus does not change with time. This can be observed in the *G*_*s*_ versus drop resting time plots that are exemplified for HeLa – water and HeLa – DMSO system shown in Fig. 13.

The reduction in *G*_*s*_ when 10% DMSO is added to the water shows that both tissues have lower resistance for interaction with the solvent once DMSO is added, and this paper quantifies this tendency for the first time. Thus, the higher reduction in *G*_*s*_ for 10% DMSO-HaCaT system shows that HaCaT substrates have lower resistance to interact with aqueous DMSO compared to HeLa. The facts that HaCaT is a skin tissue culture and the higher reduction in *G*_*s*_ for this tissue when 10% DMSO is added to the water, agree with the wider use of DMSO in dermal drug delivery methods.

## Conclusion

The interactions of water and DMSO solution to HeLa and HaCaT tissue cultures of varying rigidities was investigated. While it was known that DMSO increases the affinity of the solvent to the tissue, such knowledge was not quantitative as of yet. Here, we quantify this property of DMSO by measuring the interfacial modulus - *G*_*s*._ As expected, the interfacial modulus decreases with addition of DMSO, and we provide an exact quantification to this effect using a Centrifugal Adhesion Balance (CAB) measurements at effective zero gravity. The CAB study also shows that the drop’s lateral retention force increases with the time the drop rests on the surface (drop resting time) and eventually reaches, or clearly approaches, a plateau. This *time effect* was observed for both HeLa and HaCaT tissue cultures, and is yet another feature of the interfacial modulus which describes the resistance of a solid (or tissue) surface to interact with a contacting liquid. The higher the interaction, the more significant is the solid surface molecular re-orientation that gives rise to the interfacial modulus. The interfacial modulus is not expected to be a function of the tissue’s rigidity, and indeed we found no correlation between these properties. This study demonstrates how to quantify interactions of any tissue with any solvent (particularly pharmaceutically relevant solvents) or any tissue penetration enhancers.
